# Increase in the extracellular glutamate level during seizures and electrical stimulation determined using a high temporal resolution technique

**DOI:** 10.1186/s12868-015-0147-5

**Published:** 2015-03-13

**Authors:** Laura Medina-Ceja, Kenia Pardo-Peña, Alberto Morales-Villagrán, Jorge Ortega-Ibarra, Silvia López-Pérez

**Affiliations:** Laboratory of Neurophysiology and Neurochemistry, Department of Cellular and Molecular Biology, CUCBA, University of Guadalajara, Jalisco, Mexico; Laboratorio de Neurofisiología y Neuroquímica, Departamento de Biología Celular y Molecular, Centro Universitario de Ciencias Biológicas y Agropecuarias, Universidad de Guadalajara, Camino Ing. R. Padilla Sánchez 2100, Las Agujas, Nextipac, CP 45110 Zapopan, Jalisco Mexico

**Keywords:** 4-Aminopyridine, Bicuculline, Glutamate, Microdialysis, Novel device, Seizures

## Abstract

**Background:**

Glutamate has been measured using different methods to determine its role under normal and pathological conditions. Although microdialysis coupled with HPLC is the preferred method to study glutamate, this technique exhibits poor temporal resolution and is time consuming. The concentration of glutamate in dialysis samples can be measured *via* glutamate oxidase using the Amplex Red method.

**Methods:**

A new device has been designed and constructed to rapidly deposit dialysis samples onto a polycarbonate plate at Cartesian coordinates (every five seconds). The samples were added to an enzymatic reaction that generates hydrogen peroxide from glutamate, which was quantified using fluorescence detection. Fluorescence emission was induced by laser excitation, stimulating each spot automatically, in addition to controlling the humidity, temperature and incubation time of the enzymatic reaction.

**Results:**

The measurement of standard glutamate concentrations was linear and could be performed in dialysis samples. This approach was used to determine the effect of the convulsant drugs bicuculline and 4-aminopyridine on the extracellular glutamate concentration. Seizure activity was associated with a considerable increase in glutamate that correlated with altered EEG patterns for both drugs.

**Conclusions:**

These results indicate that this method is able to read samples with high temporal resolution, and it is easy to use compared with classical methods such as high-performance liquid chromatography, with the advantage that a large number of samples can be measured in a single experimental series. This method provides an alternative approach to determine the concentrations of neurotransmitters or other compounds that generate hydrogen peroxide as a reaction product.

## Background

Due to its important role as an excitatory neurotransmitter in the central nervous system and its role in synaptic plasticity, memory and learning processes, the concentration of extracellular glutamate (Glu) has been measured under both normal and pathological conditions [1,2]. Excess extracellular Glu produces excitotoxicity and has been associated with several neurological disorders, such as Alzheimer’s disease, stroke, neurodegeneration and epilepsy [3-6]. To better understand the role of Glu in normal or altered brain function, it is necessary to monitor this neurotransmitter at high temporal resolution, particularly when behavior or electroencephalography (EEG) activity are simultaneously evaluated [7,8]. However, although behavior and EEG activity can be studied in real time, the latency when studying the concentration of Glu depends on the utilized method.

Microdialysis is the preferred method to measure extracellular Glu concentrations, whereby samples are periodically collected (with a resolution of minutes) and quantified by high-performance liquid chromatography (HPLC). This method is reliable, and although it is considered to be the method of reference, it is frequently assumed to exhibit poor temporal resolution because a minimum volume is required to perform this analysis and a flow rate of 2.0 μl/min is generally used. Because the temporal resolution when using microdialysis typically depends on the speed of sample collection, although rapid collection improves the temporal resolution, it may also generate a bottleneck, given the challenge of measuring the large number of samples that can be obtained using this classic technique. Nevertheless, by coupling the microdialysis technique to capillary electrophoresis (CE) with laser-induced fluorescence detection (LIFD), it has been possible to measure extracellular Glu concentrations in dialysate samples at one and ten second resolution, respectively [9,10]. This procedure offers the advantage of improving the temporal resolution with respect to the classic HPLC methods, although handling such small sample volumes may be an important issue to consider due to evaporation. Another important advantage of HPLC and CE is that a group of compounds sharing similar physicochemical properties, such as monoamines or amino acids, can be separated, identified and quantified in the same sample.

Monitoring a particular neurotransmitter such as Glu at high temporal resolution using HPLC or CE can be time consuming, and considerable training is required, in addition to the cost of these devices. The recent introduction of biosensors is gradually generating additional possibilities to study rapid neurochemical changes, such as those in Glu, acetylcholine [11] and metabolic agents such as glucose and lactate [12]. These biosensors are based on the use of oxidases that generate hydrogen peroxide (H_2_O_2_), with oxidation occurring on a platinum electrode at positive potential. Biosensors can be used to monitor Glu concentrations at enhanced time resolution, allowing behavior and EEG activity to be simultaneously investigated [8,13]. Currently, biosensors are available in smaller sizes that incorporate more advanced technologies (Quanteon, LLC).

The use of biosensors opens the possibility of obtaining sufficient temporal resolution to correlate the changes in neurotransmitters with biochemical data. However, biosensors are primarily used to monitor a single neurotransmitter at a time, and in the case of acetylcholine, they do not distinguish choline from acetylcholine, although multiplexed electrodes covered with different enzymes have been used to evaluate more than one neurotransmitter, such as monoamines. Moreover, biosensors for other important neurotransmitters, particularly for GABA, remain to be developed, although the utilization of GABAse and glutamate oxidase to generate current for the electrochemical detection of a H_2_O_2_ derivate using these sets of reactions has been reported [14]. Additionally, using electrochemical biosensors for the simultaneous monitoring of neurotransmitters with measurement of low field potential results in similar signals, particularly those at low frequency; therefore, distinguishing the chemical signal from the electrical signal may be an important issue, which may be resolved through the use of a pure chemical method. Another important issue with the use of an electrochemical biosensor method is that a large potential is generally applied to generate the expected current. A decrease in sensitivity is observed that has been associated with fouling of the electrode surface due to the deposition of undesired compounds, hindering the establishment of a steady baseline for the neurotransmitter concentration. In this respect, the application of a particular coating over the electrodes has been used to inhibit or retard adhesion to the surface [15]. An alternative approach to determine neurotransmitter concentrations in biological fluids at high temporal resolution involves the use of enzymatic reactors. This approach provides new opportunities to develop and design enzymatic reactions to obtain “a derivate” that can be measured by fluorescence, luminescence and electrochemical detection systems, or even by other systems of detection. Indeed, several kits are currently available that measure Glu and acetylcholine using fluorescence (Amplex Red, Invitrogen), although these approaches require relatively large volumes of these compounds. Although the use of enzymatic reactors does not exhibit differences in specificity compared with biosensors, these approaches may offer the major advantage of preserving a more naive enzyme and its molecular properties; moreover, the enzymes are not denaturalized or restricted in a polymeric matrix such as in biosensors, which may decrease their affinity for their substrates [16]. In addition, other factors such as temperature, time incubation, and ionic strength, among other conditions, can be better controlled using these approaches.

Therefore, the objective of this study was to develop a device that utilizes the Amplex Red method to measure Glu in small dialysis samples. Dialysate samples from dialysis tubes were mixed with a similar volume of an enzymatic reactor and automatically deposited under humid conditions on a polycarbonate plate at specific Cartesian coordinates. The samples were allowed to react, and the fluorescence was subsequently measured using a charge-coupled device (CCD) detector following laser excitation of each sample. We evaluated this method in seizure models induced by the GABA_A_ receptor antagonist bicuculline and by administration of the potassium channel blocker 4-aminopyridine (4-AP), as well as through electrical stimulation. The obtained results clearly demonstrate that the enzymatic reactor can be adapted to monitor neurotransmitters in dialysate samples at high temporal resolution and with simultaneous EEG recording. This method may represent a suitable approach to avoid the separation required for chromatographic methods, and it provides a means to measure many samples in a single session, saving valuable time and reagents.

## Results

### Setup and calibration

**Figure 1:**
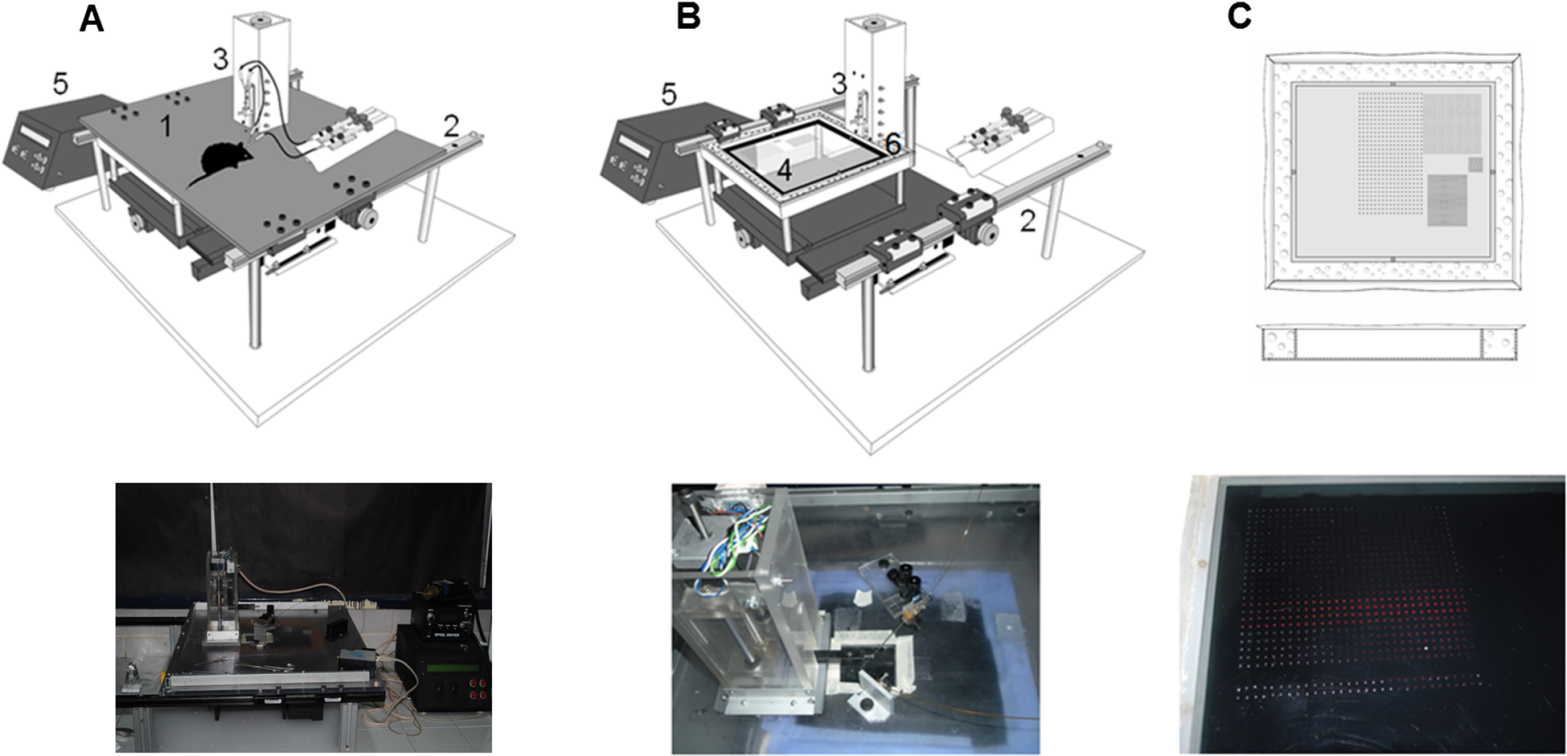
**The device developed for sampling and reading of the glutamate concentration in dialysates.** Top of **A** and **B** show the schematic device with all of its components: 1, the cover; 2, the stage movable in two axes; 3, the Z axis with the needle for sampling; 4, polycarbonate plate; 5, the control box used to program the device in terms of the number of rows and the frequency of the sampling process, as well as for reading; and 6, space for a wet sponge to create the humidity chamber when the cover slides over it. Actual images of the schematic device are shown at the bottom. **C**, Schematic and actual images (top and bottom, respectively) of fluorescence samples obtained at the end of an experiment.

The designed device constructed for these studies fulfilled the sampling and fluorescence reading requirements according to the desired protocol. The device could be satisfactorily programmed without errors, neither during deposition nor during the reading of the samples. Additionally, the humidified chamber that was coupled to the sampling stage was crucial in preventing sample evaporation, allowing the reaction to proceed despite incubation at room temperature. The incubation time (60 min) for each sample prior to reading was maintained constant, and the fiber optic arrangement enabled the light to be emitted directly over the samples and to detect the light emitted following excitation. The fiber optics was mounted through a micromanipulator to assess their exact position in each experiment. Different intensities of laser power were evaluated to obtain the best response, and 5 mW was found to be the optimal power to excite the samples and obtain the strongest fluorescence response. For the CCD-based fluorescence detector, it was possible to use the software to synchronize the time of reading (every five seconds) with the movement of the platform such that the reading could be performed automatically. Accordingly, the time required for reading depended on the total number of deposited samples. This process offers the advantage of measuring an experimental series in a single session, and using the SpectraSuite software (Ocean Optics), the analysis could be performed simultaneously during the reading. Thus, a total of 900 samples could be evaluated in approximately 75 min. A scheme of the device is presented in Figure 1.

**Figure 2:**
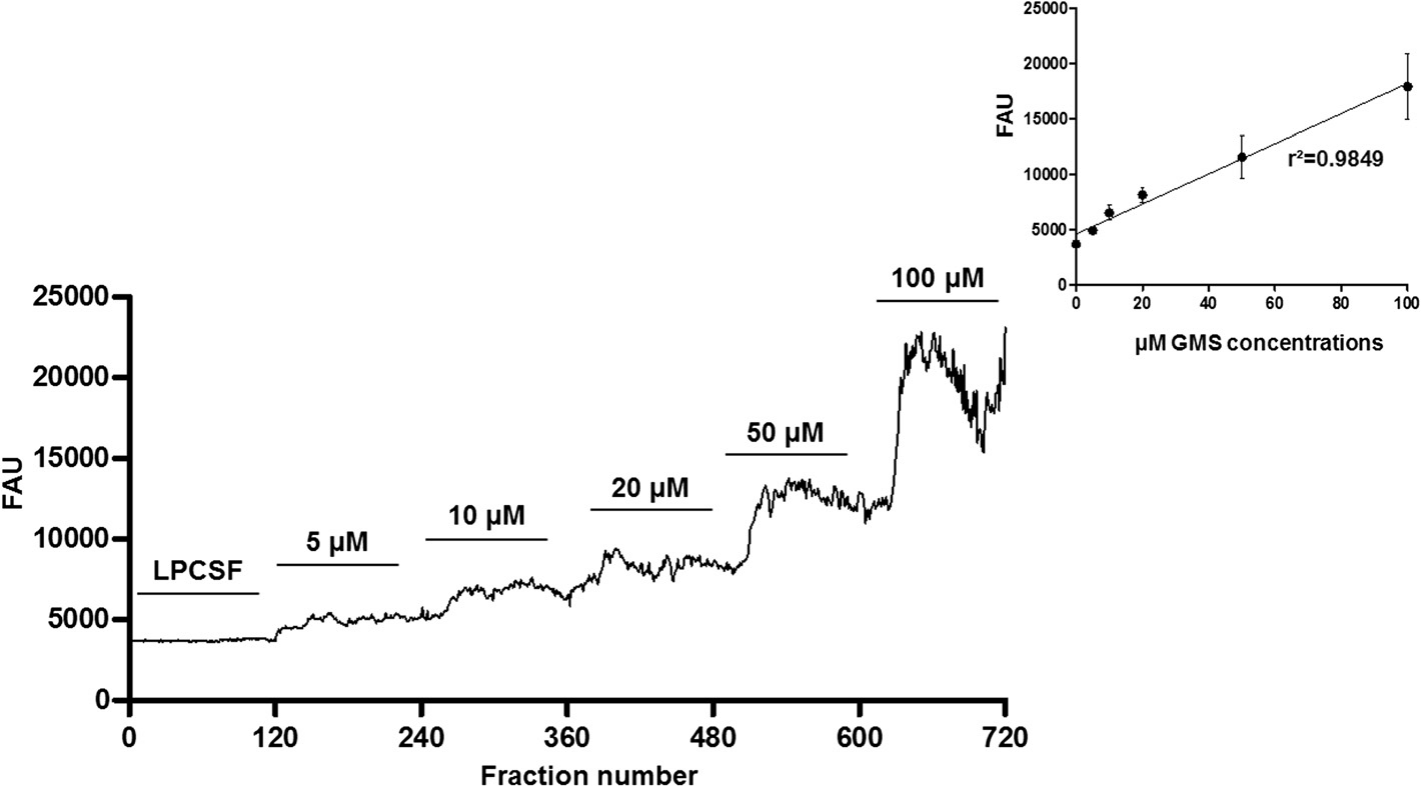
**Representative calibration curve with different concentrations of glutamate (Glu).** Glu was measured in arbitrary fluorescence units (AFU) in 720 samples collected every 5 s. The first 120 samples were analyzed without Glu, and the remaining 600 samples were analyzed with the five evaluated standard concentrations (5, 10, 20, 50 and 100 μM). The plot in the inset shows the mean (±SEM) of each concentration obtained from each experiment and the linear regression analysis of all calibration curves prior to the experiment (r^2^ = 0.98).

To evaluate the viability of the enzymes used to prepare the reactor, standard concentrations were run prior to experiments to determine calibration curves, and similar results were obtained for each calibration curve (a representative calibration curve is shown in Figure 2). These assays ensured that the reactor was in conditions to perform the experiments in animals, and a linear increase was observed in the fluorescence obtained with each increase in the standard Glu concentration. A total of 720 samples were deposited on the plate, testing 120 blank samples and 600 samples of the five evaluated standard concentrations; the value of the blank samples was subtracted from the data obtained from the experiments to calculate the actual Glu concentration. An average of the data obtained for each concentration (120 samples) from each experiment was calculated for linear regression analysis (r^2^ = 0.98). Some fluctuations appeared during the reading process that was more evident at higher concentrations of the different evaluated standards. Although the origin of these fluctuations is unclear, these fluctuations may result from mixing of the sample inside of the tubing, which may be reduced by including a mixing chamber, not excluding errors during the detection of fluorescence emission. Additional studies are necessary to confirm this speculation.

### EGG recordings and *in vivo* microdialysis studies

**Figure 3:**
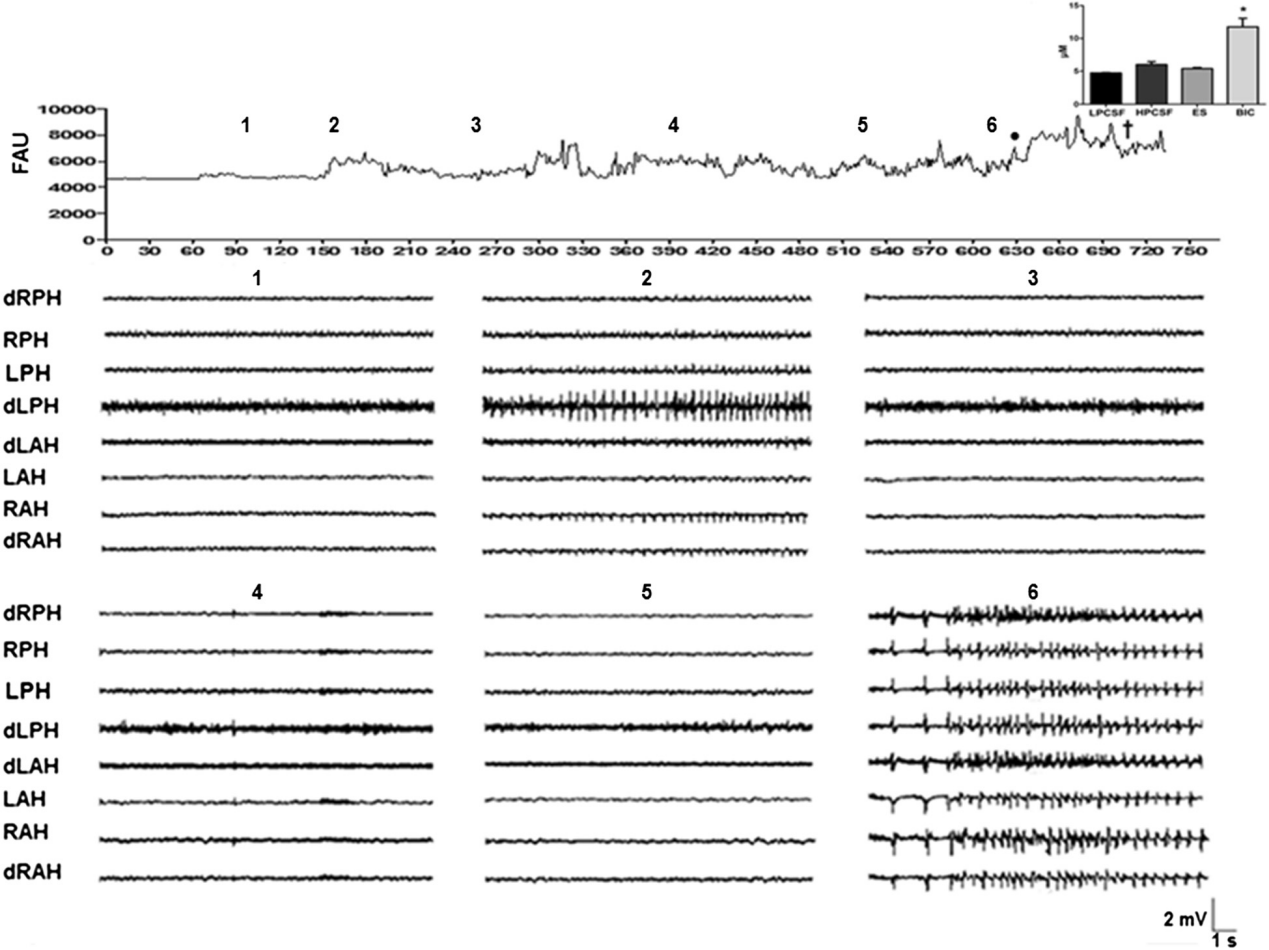
**Representative experiment in which glutamate (Glu) was simultaneously measured with EEG in the first experimental group.** The temporal course represents the Glu measured in arbitrary fluorescence units (AFU) every five seconds and the intracranial EEG recording obtained from different evaluated regions (right and left anterior hippocampus: RAH and LAH, respectively; right and left posterior hippocampus: RPH and LPH, respectively; d, deep microelectrode positioned at a 1.5 mm vertical tip separation from its pair) in 15-s segments during the administration of 1) low-potassium artificial cerebrospinal fluid (LPCSF), 2) high-potassium artificial cerebrospinal fluid (HPCSF), 3) LPCSF, 4) LPCSF + electrical stimulation (ES; duration: 0.1 ms; current: 0.5 mA every 10 s), 5) LPCSF, and 6) LPCSF + bicuculline (BIC, 8 mg/kg, i.p.). The plot in the inset corresponds to the mean (±SEM) of the Glu concentration in μM during the different evaluated protocols (n = 5). The value in each bar was obtained by averaging 10 min of sampling (120 samples). •Onset of epileptiform activity; †time of death of the animal while seizing; *p < 0.05 *vs*. other protocols.

To evaluate the different protocols, 5 rats were used, each of which exhibited normal electrical activity prior to testing. This activity was characterized by slow physiological waves of low amplitude and frequency (LPCSF, Figure 3), and an increase, particularly in amplitude, was observed in most evaluated regions following HPCSF administration to the RPH (RAH 570.3 ± 233, LAH 705.6 ± 234, RPH 1069.3 ± 422 and LPH 1497.2 ± 726 μV). In contrast, only a small increase in frequency was detected (RAH 4.3 ± 0.7, LAH 4.0 ± 0.8, RPH 3.4 ± 0.6 and LPH 3.6 ± 0.6 Hz), and both of these parameters returned to the basal state during subsequent LPCSF administration. In contrast to the changes produced by HPCSF administration, both the amplitude and the frequency remained close to the basal level of electrical activity during electrical stimulation in the four analyzed regions (RAH 547.6 ± 146, LAH 510.7 ± 116, RPH 514.2 ± 188 and LPH 561.4 ± 90 μV; RAH 4.6 ± 0.4, LAH 3.6 ± 0.6, RPH 2.9 ± 0.7 and LPH 3.0 ± 0.7 Hz), and similar basal electrical activity persisted following LPCSF administration.

Bicuculline administration produced intense epileptiform activity in all evaluated regions (Figure 3), with an average latency for the appearance of epileptiform discharges of 2.6 ± 0.1 min. The maximum amplitude of the first epileptiform discharge was observed in the RPH (2867.6 ± 567 μV, p < 0.001) and the minimum amplitude in the LAH (2275.2 ± 477 μV, p < 0.001), whereas the maximum frequency was observed in the LPH (9.0 ± 2.7 Hz, p < 0.001) and the minimum frequency in the RAH (5.2 ± 2.0 Hz, p < 0.001). Although a small decrease in the amplitude of epileptiform discharges in all of the evaluated regions (RAH 1445 ± 339, LAH 1604.6 ± 436, RPH 1804.2 ± 304 and 1542 ± 393 μV) was observed at the end of the experiment, a general increase in the frequency of this epileptiform activity was also observed (RAH 16.9 ± 6.9, LAH 17.4 ± 7.0, RPH 8.1 ± 3.1, and LPH 10.2 ± 3.0 Hz).

The neurochemical data revealed a basal glutamate level of 4.7 ± 0.06 μM. During HPCSF administration, a small discrete increase in Glu of 27.1% (6.0 ± 0.44 μM) was observed that was correlated to the small increment in the amplitude and frequency of electrical activity (Figure 3). The stimulation protocol also produced an increase in Glu of 14.2% (5.4 ± 0.18 μM) but no pronounced changes in electrical activity. In contrast, a strong 97.6% increase in Glu was observed during the first epileptiform discharge induced by bicuculline administration (9.3 ± 0.38 μM, Figure 3). In addition, an additional increase in Glu was evident and persisted throughout the period of seizures before the death of the animals (149% with respect to the baseline level, 11.7 ± 1.3 μM; p = 0.0017). This increase was coincident with the period of constant high amplitude and frequency epileptiform activity induced by bicuculline (Figure 3).

**Figure 4:**
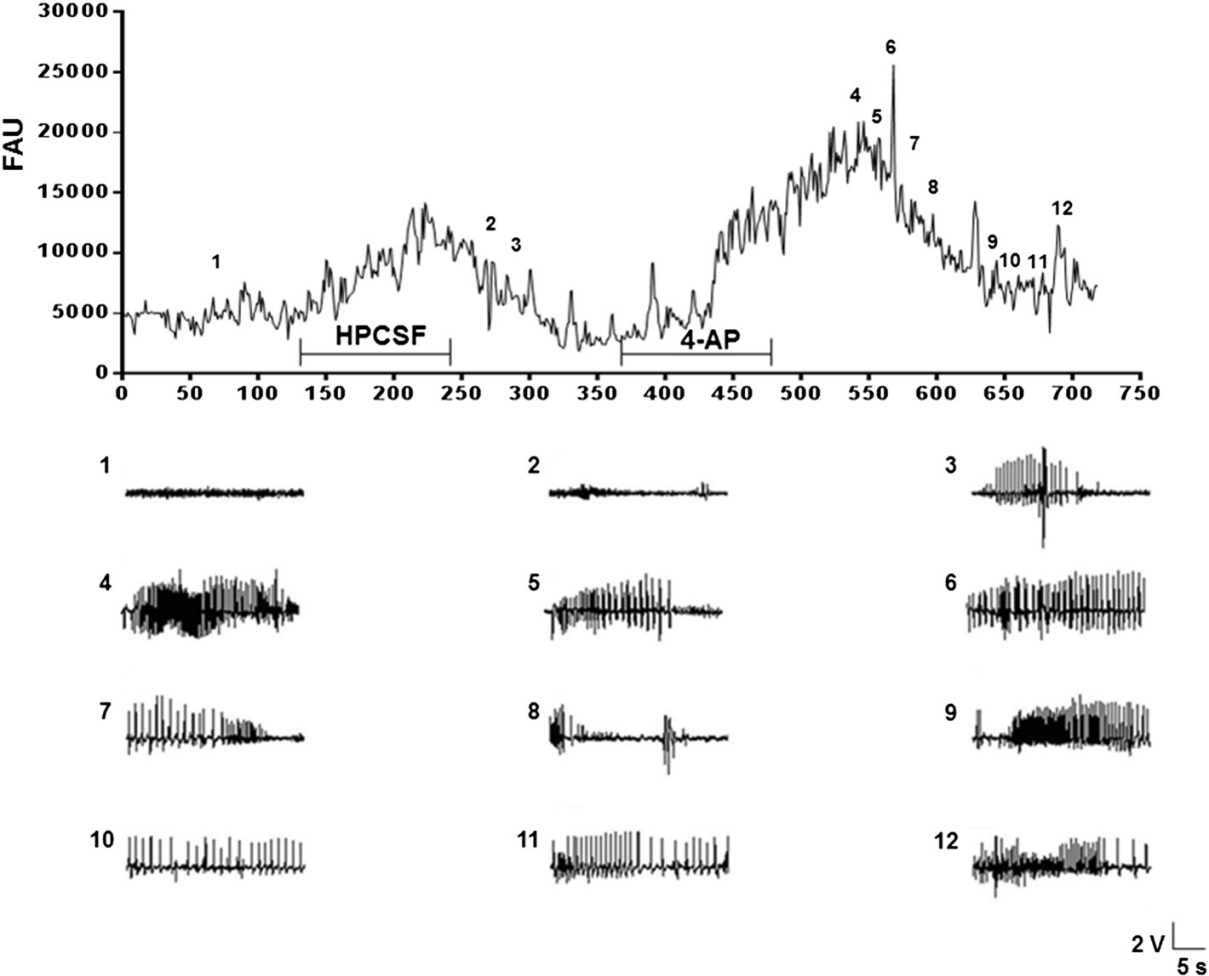
**Representative experiment in which glutamate (Glu) was simultaneously measured with EEG in the second experimental group.** The plot represents the temporal course for glutamate (Glu) levels in arbitrary fluorescence units (AFU) observed every five seconds throughout a representative experiment (n = 4), and the EEG recording in segments of 30 s during administration of low-potassium artificial cerebrospinal fluid (1), high-potassium artificial cerebrospinal fluid (HPCSF, 2 and 3 with interictal spikes), and 4-aminopyridine (4-AP; administered over 10 min through the microdialysis probe, 4–12; 25 mM).

In the animals that were administered 4-AP, the amplitude and frequency of activity was low during basal activity (RFC 154 ± 29, LFC 192 ± 43, ROC 217 ± 35, LOC 248 ± 33 and RPH 467 ± 41 μV; RFC 1.7 ± 0.4, LFC 2.2 ± 0.1, ROC 2.5 ± 0.3, LOC 2.3 ± 0.2 and RPH 1.7 ± 0.2 Hz). Indeed, during HPCSF administration, only one 30-s period with interictal spikes was observed (RFC 381, LFC 579, ROC 614, LOC 795 and RPH 4120 μV), and the frequency did not differ from the basal electrical activity (1.5-3.5 Hz). The administration of 4-AP produced an initial epileptiform discharge after 120 s, with a marked increase in amplitude that was primarily evident in the RPH (RFC 98 ± 10, LFC 152 ± 9, ROC 371 ± 10, LOC 221 ± 14 and RPH 2702 ± 104 μV; RFC 3.0 ± 0.2; LFC 2.0 ± 0.3, ROC 2.0 ± 0.3, LOC 3.0 ± 0.7 and RPH 2.0 ± 0.7 Hz). Subsequent epileptiform activity was characterized by polyspike trains and synchronized spike wave activity associated with high amplitudes and frequencies (RFC 209 ± 30, LFC 189 ± 20.8, ROC 370 ± 52, LOC 326 ± 33.2 and RPH 1925 ± 155.6 μV; RFC 3.0 ± 0.6; LFC 5.0 ± 1.3, ROC 5.0 ± 1.8, LOC 4.0 ± 1.1 and RPH 5.0 ± 1.5 Hz). These changes were more evident in the RPH area, close to the point of 4-AP administration (Figure 4).

These rats had a basal Glu concentration of 4.4 ± 0.07 μM, which increased significantly during HPCSF perfusion to 18.4 ± 0.05 μM. This alteration was associated with the interictal spikes observed in the EEG recordings. Moreover, 4-AP administration was correlated to an increase of 47.7 ± 0.76 μM in the RPH associated with the strong convulsive behavior and epileptiform activity that appeared in all of the evaluated regions. Intermittent Glu alterations were observed throughout the experimental procedure and were correlated to alterations in the EEG and convulsive behavior (Figure 4). All of the neurochemical data were correlated to the EEG following correction for the dead time latency (7 min).

## Discussion

We have developed a device for the rapid sampling and measurement of small volumes of microdialysis samples (Innova Cielec, SA de CV, Tonalá, Jalisco, México). This device can be programmed in several ways, such as based on the time and number of samples to be deposited on the platform, such that large numbers of samples can be rapidly analyzed (in this case, greater than 700 samples in a single 60-min session). The sampling and reading procedure is highly reproducible and does not produce mechanical or electronic errors. The humidity chamber used with this device was sufficient to prevent sample evaporation, allowing the samples to remain in solution throughout the experiment and for several additional hours. To evaluate its functionality, calibration curves were determined, and the average linear regression analysis was 0.96 in most of the sessions. However, apparent noise was observed with increasing Glu concentration, which may be due to the filter used (grid window); improved filtering of light emission may reduce this noise. With these functional characteristics, the device could be used to analyze several neurotransmitters (Glu in this case) that can be measured with enzymatic reactors that generate H_2_O_2_ as a reaction product.

In the present study, this device was used to measure changes in the Glu concentration correlated to EEG recordings of rats treated with bicuculline or 4-AP in small dialysis samples at high temporal resolution. The fluctuations observed during calibration increased with higher Glu concentrations. As previously mentioned, these fluctuations may result from mixing of the sample inside of the tubing; including a mixing chamber may aid in reducing these fluctuations, although we cannot exclude the possibility of errors during the fluorescence readings. The use of an optical shutter to block undesired light between each measurement may aid in preventing these fluctuations, which limit the ability to detect concentrations less than 1.0 μM. Further optimization of the performance of this instrument would improve the limit of detection. The baseline Glu concentration (in μM) is similar to the concentration obtained using HPLC methods with both electrochemical and fluorometric detection (2.8 μM/1.5 μM). This small difference between these two methods may be due to the cut-off of the dialysis membrane. For the HPLC-based methods, a 6 kDa cut-off membrane was used, and, particularly for the lowest concentration determined by fluorometric detection, the experiments were performed under anesthesia; in the present set of experiments, a 20 kDa cut-off membrane was used, which results in better recovery, and the animals were not under anesthesia [7,17]. Although the method used in this study does not have the limit of detection of electrochemical or fluorescence HPLC methods, it demonstrates sufficient sensitivity to monitor a clear baseline Glu concentration during normal physiological activity; considering a 3:1 signal-to-noise ratio, the limit of detection for determination of the Glu concentration in each sample was 1.0 μM. Additionally, the signal fluctuation present throughout the experimental procedure may be due to the form of the samples on the polycarbonate plate, *i.e*., they are not flat or embedded in a polymeric matrix, which permits enhanced fluorescence detection. Additional studies on instrument optimization are currently ongoing to prevent these fluctuations. The results indicated that discrete increases in the Glu concentration (27.1%) were correlated with a small increment in the amplitude and frequency of electrical activity, particularly using the HPCSF protocol in rats that received bicuculline. Moreover, when interictal activity was present during HPCSF administration in the animals that received 4-AP, a significant increase in the Glu concentration (300%) was observed. These results are consistent with our previous studies [6,7], in which a threefold increase in the Glu concentration was provoked by HPCSF administration. In addition, weak electrical stimulation (duration: 0.1 ms; current: 0.5 mA every 10 s/10 min) to the right angular bundle that did not produce pronounced changes in electrical activity was sufficient to increase the Glu concentration (14.2%). This result may indicate that the release of Glu evoked by electrical stimulation is not sufficiently strong to synchronize the electrical activity of a neuronal population and produce significant changes in EEG recordings. This finding is in contrast to the correlation between low-frequency stimulation-evoked Glu currents and the slopes of the field excitatory postsynaptic potentials [18] (fEPSPs). Conversely, bicuculline administration produced a significant increase in the Glu concentration (97.6%) that was correlated with the first epileptiform discharge and persisted throughout the experimental procedure (149%), together with the epileptiform activity. Indeed, bicuculline administration through different routes preceded the increase in extracellular Glu. For example, the Glu concentration increased approximately 100% in the rat hypothalamus following intracerebroventricular administration of 0.3 nmol bicuculline in the absence of genetic epileptic predisposition [19], whereas the infusion of bicuculline (100 μM) through a microdialysis probe increased Glu levels by 46% in the ventral hippocampus [20]. In both of these studies, a traditional HPLC system coupled to electrochemical detection was used to measure microdialysis samples collected every 20 min, with offline analysis. Neither of these studies adequately correlated the EEG activity with the neurochemical data. In the kainate model of epilepsy, a 2- to 6-fold increase in Glu was observed in association with the frequency and magnitude of electrographic seizures in the hippocampus [21]. Nevertheless, this study used 2 min microdialysis samples, fluorometric detection and offline analysis, a methodology that does not permit a detailed correlation with the precise onset of the electrographic seizures. Other studies demonstrated bicuculline to be a competitive antagonist of the GABA binding site in the GABA_A_ receptor, as well as producing prolonged Ca^2+^ action potentials and blockade of K^+^ channels [22], effects that produce seizures. During seizures, bicuculline was also found to induce high-amplitude and frequency polyspike discharges, followed by clonic paroxysmal activity of lower frequency [23]. These results are consistent with the epileptiform activity observed in the present study. The microdialysis procedure is generally related to the extracellular neurotransmitter concentration, which may originate from synaptic activity and reverse Glu transport mediated by transporters localized both in neurons and in glia [24]. Additional experiments should be conducted to determine the source of Glu, such as using tetrodotoxin to block synaptic release. However, during HPCSF and bicuculline administration, significant increases in Glu were found that may be related to synaptic activity, as previously discussed [20,25]. Although probe insertion produces significant tissue damage that results in an immediate increase in the Glu concentration, in this study, an increase due to probe damage can be excluded because the probe was allowed to stabilize to reach a baseline level prior to the experiments. Therefore, the measured Glu primarily originates from the intact tissue surrounding the microdialysis probe.

We also evaluated the convulsive effect of 4-AP to demonstrate the correlation between Glu release and epileptiform activity measured with this novel device. As observed for animals treated with bicuculline, animals treated with 4-AP exhibited a close correlation between increased Glu and electrical activity. During HPCSF administration, an increase in Glu levels was correlated with interictal spike periods, whereas during 4-AP administration, the Glu concentration peaked in association with the first epileptiform discharge. In this experimental group, the observed Glu increase may originate from synaptic terminals because 4-AP is known to block potassium channels, increasing the intracellular calcium concentration and allowing neurotransmitter release [26]. Previous studies attempted to correlate electrical activity with Glu levels following 4-AP administration [7,27,28], with high Glu concentrations found during electrographic seizures. However, the timescale over which the dialysis samples were collected was long, and in the best case, the samples were collected every 60 s, rendering it difficult to correlate them to physiological events.

Although microdialysis is the traditional technique used to measure Glu concentrations during brain activity, its low temporal resolution, with minimal temporal intervals of one or two minutes, renders it difficult to correlate rapid brain electrical activity with the neurotransmitter concentration. Although several studies have attempted to measure Glu levels at higher time resolution in small volumes [9,10,29], these studies typically required expensive equipment and involve time-consuming procedures, relying on specialized techniques that require specific training to perform. Although biosensors are currently widely used and their use is emerging as a popular method to determine neurotransmitter concentrations, additional effort is ongoing to obtain more reliable data. Optimization of the techniques presented in this study (*i.e*., electrochemical and optical biosensors combined with the rapid sampling of microdialysis samples) may offer a better means of establishing a correlation between electrophysiological and biochemical activity.

## Conclusions

The device we have developed and present herein provides a good alternative to measure Glu concentrations with sufficient temporal resolution for correlation of rapid electrical activity with neurochemical changes in samples collected every five seconds. This device is less expensive to use and easy to handle compared with other devices that may be capable of achieving a similar resolution. Using this device, we correlated increases in the Glu concentration with the interictal spike period during HPCSF administration or with the first epileptiform discharge provoked by bicuculline or 4-AP. Although these results are consistent with other studies demonstrating high Glu levels during epileptiform activity, it has not been previously possible to correlate these events this closely, and previous results were based on the accumulation of Glu during longer microdialysis collection times.

## Methods

### Enzymatic glutamate assay and the developed device

The assay used in this study has been shown to provide a sensitive means to detect extracellular Glu and is a tested method utilized in commercially available kits to determine the concentrations of several compounds using Amplex Red (Invitrogen, San Diego, CA, USA). Briefly, Glu is oxidized by glutamate oxidase to produce α-ketoglutarate, NH_3_ and H_2_O_2_. The reaction includes L-alanine and L-glutamate–pyruvate transaminase to regenerate L-Glu by transamination of α-ketoglutarate, amplifying the H_2_O_2_ produced by permitting multiple cycles of the initial reaction. The generated H_2_O_2_ is measured with Amplex Red (10-acetyl-3,7-dihydroxiphenoxazine), which is a fluorogenic probe that reacts with H_2_O_2_ in the presence of horseradish peroxidase in a 1:1 stoichiometric ratio, producing resorufin [30,31]. Resorufin fluoresces at 590 nm when excited at 560 nm, and this fluorescence can be readily measured in small cuvettes [28].

Standard solutions were prepared by diluting Glu in artificial cerebrospinal fluid (aCSF) containing the following salts (in mM): NaCl (125); KCl (2.5); NaH_2_PO_4_•H_2_O (0.90); Na_2_HPO_4_ (5); MgCl2•6H_2_O (1.0); and CaCl_2_ (2.2). An enzymatic reactor was prepared by mixing glutamate oxidase (0.1 U/ml), horseradish peroxidase (0.25 U/ml), L-alanine (200 μM), and L-glutamate–pyruvate transaminase (0.1 U/ml). All enzymes and reagents were obtained from Sigma-Aldrich (St. Louis, MO, USA), and Amplex Red (100 μM) was obtained from Invitrogen (San Diego, CA, USA). Calibration curves were obtained by mixing the standard solutions with the enzymatic reactor through a “Y” connector needle attached to a linear actuator (“Z” axis). This needle was equipped with a spring buffer that permitted the samples to be placed onto the plate without the needle bending and reaching the surface. One inlet was used to feed the reactor solution, and a second inlet was used for the standard solution or dialysate from the animals. Two microdialysis syringe pumps (Bioanalytical Systems, Inc., West Lafayette, USA) were used to simultaneously administer the reactor and the standard solution or dialysis samples, and the mixture exiting from the “Y” connector was periodically deposited at Cartesian coordinates onto a polycarbonate plate placed over a motorized X, Y stage. Customized hardware and software were developed to program the number of rows and columns to be deposited on the plate, as well the frequency of sampling, in these experiments every five seconds (Innova Cielec SA de CV, Tonalá, Jalisco, México). Individual syringes were filled with several standard solutions, and two rows of 30 samples were deposited on the plate. The following consecutive concentrations of Glu were evaluated using a liquid switch: 5, 10, 20, 50 and 100 μM. Because the flow rate of both syringes was 2.5 μl/min, the volume deposited in each sample was approximately 400 nl every five seconds. To prevent evaporation of these small volumes, a humidified chamber was constructed over the plate, permitting mobility in the Z axis to deposit the respective samples. With this setup, it was possible to maintain the humidity of the samples for long periods of time, even up to 48 h (Figure 1A and B). This procedure was performed prior to each *in vivo* experiment.

To determine the Glu content in each deposited sample, a fluorescence reading system was coupled to this device that consisted of a CCD detector (Ocean Optics). To excite the samples, a laser light source was used (561 nm wavelength; Opto Engine LLC, China), conducting the light to the sample through fiber optics (400 μm in diameter) placed approximately 1 mm above the sample. The distance and laser intensity were set at 5 mW to excite and obtain an adequate fluorescence signal. To detect the fluorescence intensity, another optical fiber (600 μm) was placed at a similar distance but at 90° with respect to the excitation source. Once the emission was detected and filtered (590 nm wavelength), the fluorescence was sent to the detector (Figure 1C). The measured fluorescence intensity was used to obtain the calibration curves and evaluate the reliability of this procedure. Additionally, the fluorescence intensity obtained for the samples was extrapolated to the values obtained in the calibration curves. Fluorescence values of the standards and samples that were larger than three times the standard deviations obtained at zero Glu concentration (blank) were considered actual concentrations and were used to determine the limit of detection (1.0 μM).

### Animals and surgery

All experimental procedures were designed to minimize animal suffering and the total number of animals used. All rats were maintained in individual cages in a temperature-controlled room on a 12 h light/dark cycle with access to food and water *ad libitum*. This protocol conformed to the Rules for Research in Health Matters (Mexican Official Norms NOM-062-ZOO-1999, NOM-033-ZOO-1995) and was approved by the local Animal Care Committee.

Extracellular Glu was measured in nine animals; the first group consisted of five adult male Wistar rats (250–350 g) following different experimental protocols, such as the effect of a high-potassium solution, electrical stimulus and bicuculline (i.p. administration), and a second group of four animals was used to measure Glu following 4-AP administration (reverse microdialysis procedure). The animals were anesthetized with isoflurane in 100% O_2_ and secured in a Stoelting stereotaxic frame with the incisor bar positioned at −3.3 mm. A pair of tungsten microelectrodes (60 μm in diameter) was implanted in the right angular bundle to stimulate the perforate pathway (AP −7.00 mm from bregma; ML −4.00 mm; DV −2.5 mm from the superficial neocortex), with a vertical separation of 0.5 mm between the tips. Two fixed microelectrodes (bipolar microelectrodes) were assembled with the same characteristics as the stimulation microelectrodes but with a 1.5 mm vertical tip separation, and they were implanted in each evaluated region, as follows: left and right posterior hippocampus (LPH, RPH: AP −5.0 mm from bregma; ML ± 5.0 mm; DV −5.5 mm), left and right anterior hippocampus (LAH, RAH: AP −3.5 mm; ML ± 2.00 mm; DV −4.0 mm). A stainless steel guide cannula (0.5 mm internal diameter) was implanted into the right posterior hippocampus (AP −5.0 mm; ML −5.0 mm; DV −5.5 mm), and this cannula was used to insert the dialysis probe. All microelectrodes were attached to a socket connector and, together with the guide cannula, were fixed to the skull with acrylic dental cement. To evaluate the effect of the convulsant drug 4-AP, a different surgical procedure was performed on four animals, attaching four superficial electrodes to the right and left frontal (RFC, LFC) and occipital (ROC, LOC) cerebral cortex and introducing a bipolar deep microelectrode into the guide cannula in the RPH.

### Drug administration and microdialysis

Three days after surgery, a CMA-12 microdialysis probe (0.5 mm in diameter, 3 mm in length and 20 kDa cut-off) was inserted into the hippocampus. The probe was flushed with HPLC-quality water at a flow rate of 10 μl/min for 5 min prior to insertion, after which the fluid solution was switched to a filtered, low-potassium, artificial cerebrospinal fluid (LPCSF) adjusted to pH 7.4 with CO_2_ that contained the following (in mM): NaCl (125), KCl (2.5), NaH_2_PO_4_ (0.9), Na_2_HPO_4_ (5), MgCl_2_ (1), and CaCl_2_ (2.2). Following insertion, the probe was allowed to equilibrate for at least 2 h at a flow rate of 2.5 μl/min, followed by simultaneous EEG recording and fraction collection. Samples were collected every five seconds and mixed with an equivalent volume of the enzymatic reactor throughout the experimental procedure, and the basal Glu concentration was determined in each experiment from the first 120 samples. The perfusion solution was then changed to a high-potassium solution (HPCSF, 100 mM KCl in aCSF with a reduced NaCl concentration to adjust the osmolarity), which was flushed through the probe for ten minutes at the same flow rate. The LPCSF was then flushed through the probe for an additional 10 min prior to application of the electrical stimulation for 10 min (duration: 0.1 ms; current: 0.5 mA every 10 s), and the probe was once again flushed with LPCSF for 10 min prior to administration of bicuculline (8 mg/kg, i.p.; antagonist of the GABA_A_ receptor (Sigma-Aldrich, St. Louis, MO, USA)).

In another series of experiments, a similar protocol was followed without testing the electrical stimulation, in which after a 2-h recovery period, HPCSF and 4-AP (25 mM) were administered through the microdialysis probe for 10 min each, with administration of LPCSF in between. To correlate the biochemical changes with the EEG electrical activity, it was necessary to determine the dead latency time, which was calculated *in vitro* by immersing the same microdialysis probe inside a beaker that contained a known Glu concentration and collecting consecutive samples, as previously described, until a clear increase in signal fluorescence could be measured.

### EEG recording

Intracranial EEG recordings were obtained using a Grass 7D polygraph with eight amplifiers. Electrical stimulation was performed using a Grass (S44) stimulator with the following parameters: high band filter of 0.1 Hz and low band filter of 3 kHz with a sensitivity 75 μV/cm, acquisition in each channel of 3 kHz, and a precision of 12 bit. The data were stored on external hard disks for subsequent analysis. The electrical signals were analyzed on a Macintosh A1048 computer using AcqKnowledge 3.9.1 software from BIOPAC Systems (California, USA). The parameters of the analyzed electrical signals were the amplitude and frequency (mean ± SEM) in periods of 15 s during the different chemical and electrical protocols in the first set of experiments, and periods of 5 s were analyzed to evaluate the effect of 4-AP administration.

### Histological evaluation

At the end of each experiment, the placement of the guide cannula was verified. The animals were anesthetized with sodium pentobarbital and transcardially perfused with 100 ml of normal saline (0.9%) in 0.12 M buffer/CaCl_2_, followed by 300 ml of 4% paraformaldehyde in 0.12 M buffer/CaCl_2_ (pH 7.3). Coronal sections (50 μm thick) of the animal’s brain were stained with cresyl violet, and animals in which the cannula reached the exact required position were included in the study.

### Data analysis

The results are expressed as the means (±SEM), and comparisons were made between different protocols using the GraphPad Prism 5 statistical software package by applying a one-way analysis of variance (ANOVA) with Dunnett’s and Tukey’s *post hoc* tests. Statistical significance was considered at p < 0.05.

## Abbreviations

4-AP: 4-Aminopyridine
CE: Capillary electrophoresis
EEG: Electroencephalography
Glu: Glutamate
H_2_O_2_: Hydrogen peroxide
HPCSF: High-potassium artificial cerebrospinal fluid
HPLC: High-performance liquid chromatography
LAH: Left anterior hippocampus
LFC: Left frontal cortex
LIFD: Laser-induced fluorescence detection
LOC: Left occipital cortex
LPCSF: Low-potassium artificial cerebrospinal fluid
LPH: Left posterior hippocampus
RAH: Right anterior hippocampus
RFC: Right frontal cortex
ROC: Right occipital cortex
RPH: Right posterior hippocampus
